# A prospective trial of elective extubation in brain injured patients meeting extubation criteria for ventilatory support: a feasibility study

**DOI:** 10.1186/cc7112

**Published:** 2008-11-10

**Authors:** Edward M Manno, Alejandro A Rabinstein, Eelco FM Wijdicks, Allen W Brown, William D Freeman, Vivien H Lee, Stephen D Weigand, Mark T Keegan, Daniel R Brown, Francis X Whalen, Tuhin K Roy, Rolf D Hubmayr

**Affiliations:** 1 Department of Neurology, Mayo Clinic College of Medicine, 200 First Street SW, Rochester, MN 55905, USA; 2 Department of Physical Medicine and Rehabilitation, Mayo Clinic College of Medicine, 200 First Street SW, Rochester, MN 55905, USA; 3 Department of Biostatistics, Mayo Clinic College of Medicine, 200 First Street SW, Rochester, MN 55905, USA; 4 Department of Anesthesiology, Mayo Clinic College of Medicine, 200 First Street SW, Rochester, MN 55905, USA; 5 Department of Neurology, Mayo Clinic College of Medicine, 4500 San Pablo Road, Jacksonville, FL 32224, USA

## Abstract

**Introduction:**

To assess the safety and feasibility of recruiting mechanically ventilated patients with brain injury who are solely intubated for airway protection and randomising them into early or delayed extubation, and to obtain estimates to refine sample-size calculations for a larger study. The design is a single-blinded block randomised controlled trial. A single large academic medical centre is the setting.

**Methods:**

Sixteen neurologically stable but severely brain injured patients with a Glasgow Coma Score (GCS) of 8 or less were randomised to early or delayed extubation until their neurological examination improved. Eligible patients met standard respiratory criteria for extubation and passed a modified Airway Care Score (ACS) to ensure adequate control of respiratory secretions. The primary outcome measured between groups was the functional status of the patient at hospital discharge as measured by a Modified Rankin Score (MRS) and Functional Independence Measure (FIM). Secondary measurements included the number of nosocomial pneumonias and re-intubations, and intensive care unit (ICU) and hospital length of stay. Standard statistical assessments were employed for analysis.

**Results:**

Five female and eleven male patients ranging in age from 30 to 93 years were enrolled. Aetiologies responsible for the neurological injury included six head traumas, three brain tumours, two intracerebral haemorrhages, two subarachnoid haemorrhages and three ischaemic strokes. There were no demographic differences between the groups. There were no unexpected deaths and no significant differences in secondary measures. The difference in means between the MRS and FIM were small (0.25 and 5.62, respectively). These results suggest that between 64 and 110 patients are needed in each treatment arm to detect a treatment effect with 80% power.

**Conclusions:**

Recruitment and randomisation of severely brain injured patients appears to be safe and feasible. A large multicentre trial will be needed to determine if stable, severely brain injured patients who meet respiratory and airway control criteria for extubation need to remain intubated.

## Introduction

More than 200,000 patients per year require mechanical ventilation primarily for neurological reasons based on rates of endotracheal intubation for patients with ischaemic and haemorrhagic stroke, head trauma and subarachnoid haemorrhage [1-6]. The direct and indirect costs of caring for head trauma patients alone is greater than 60 billion dollars annually in productivity losses and lifetime medical costs [3-6]. Improving outcome in mechanically ventilated brain injured patients would have significant medical and economic implications.

Pulmonary complications may be reduced by early extubation, for example by decreasing the rate of nosocomial pneumonia [7,8]. Thus, identifying the optimal timing of extubation in a population of brain injured patients should improve outcome and shorten length of stay in hospital.

Brain injured patients with compromised levels of consciousness are usually intubated primarily for concerns of airway maintenance and not for respiratory issues. Dogma mandates that patients with Glasgow Coma Scores (GCS) of 8 or less need to be or remain intubated to 'protect' the airway from aspiration [9,10]. However, a recent prospective study evaluating a cohort of brain injured patients found that delaying extubation based solely on a patients' level of consciousness led to an increase in the rate of nosocomial pneumonia, hospital length of stay and worse clinical outcome [11].

The authors stated that their analysis justified conducting a randomised controlled trial of early extubation in brain injured patients [11]. We assessed the feasibility of performing this study by designing a pilot study of mechanically ventilated patients with brain injury intubated solely for airway protection randomised to early or delayed extubation. The purpose was to gain an insight into patient safety concerns and to obtain broad estimates of the sample size calculations needed for a larger study.

## Materials and methods

The eligible study population consisted of all intubated patients admitted to the neurological intensive care unit (ICU) at Saint Mary's Hospital in Rochester, Minnesota. Daily screening of potential patients occurred during morning rounds in the neurological ICU by one of the study investigators. Patients were assessed for the need for continued endotracheal intubation and were considered potential candidates for the trial if they were intubated solely because of a GCS of 8 or less. Enrollment data included routine laboratory and respiratory profiles obtained for mechanically ventilated patients in the neurological ICU.

Enrollment criteria included: resolution or improvement of any pulmonary process requiring mechanical ventilation (such as congestive heart failure or pneumonia); adequate gas exchange, as indicated by a ratio of the partial pressure of arterial oxygen (PaO_2_) to the fraction of inspired oxygen (FiO_2_) above 200 with a positive end-expiratory pressure of less than 6 cm of water; adequate ventilation as indicated by a PaCO_2 _less than 45 torr or a pH between 7.35 and 7.45 if the PaCO_2 _was less than 45 torr in a patient with known chronic obstructive pulmonary disease; respiratory rate to tidal volume ratio less than 105; core body temperature less than 38°C; haemoglobin more than 8 g/dL; and no sedative medications for the previous two hours.

Neurological requirements included: GCS of 8 or less; intracranial pressure less than 15 cm of water; and a cerebral perfusion pressure more than 60 mmHg for patients with intracranial pressure monitors.

In addition to the above criteria, the responsible attending physician would have to agree that the patient was in a stable condition and was ready for extubation.

Exclusion criteria included: age younger than 18 years; lack of informed consent by the patient's surrogate; dependence on mechanical ventilation for at least two weeks before enrollment; presence of tracheostomies; intubation instituted for therapeutic hyperventilation; planned surgical or radiological intervention within the next 72 hours; anticipated neurological or medically worsening conditions (such as development of cerebral oedema or vasospasm); and intubation for airway preservation due to airway oedema (cervical neck injuries or surgery) as opposed to airway protection.

Written informed consent was obtained from the patient's surrogate if the patient met eligibility requirements. Enrolled patients underwent a 30 minute T-piece trial with no continuous positive airway pressure to evaluate readiness for extubation. The trial was discontinued if any of the following were noted: respiratory rate of more than 35 breaths per minute for at least five minutes; arterial saturation below 90% for two minutes; heart rate more than 140 beats per minute; sustained changes in heart rate of 20% in either direction; systolic blood pressure higher than 180 mmHg or lower than 90 mmHg; and a notable increase in agitation or diaphoresis.

Patients who passed a spontaneous breathing trial were evaluated using the modified Airway Care Score (ACS) to assess their ability to control their respiratory secretions (Table 1) [11].

**Table 1:** Grading for the Airway Care Score.

**Grading**	**Cough to suction**	**Sputum quantity**	**Sputum character**	**Sputum viscosity**	**Suctioning frequency**
0	Vigorous	None	Clear	Watery	> 3 hours

1	Moderate	1 pass	Tan	Frothy	Every 2 to 3 hours

2	Weak	2 passes	Yellow	Thick	Every 1 to 2 hours

3	None	≥ 3 passes	Green	Tenacious	< Every 1 hour

Passes refers to number of passes of a suctioning catheter that is required to clear the endotracheal tube of secretions. The total score is the summation of all grades.

The ACS was assessed by an ICU consultant and either the nurse or the respiratory therapist who were caring for the patient or both. ACS assessors were blinded to the other ACS assessments. Kappa values were calculated for ACS assessment between physician and nurse, and physician and respiratory therapist. Differences in ACS assessment were subsequently resolved by consensus. If the ACS was more than 7, enrollment was delayed and enrollment criteria were reassessed 12 hours later.

Patients who passed the T-piece trial and ACS assessments were eligible for randomisation. A randomised block design was utilised to assign 16 patients either into a treatment group that was extubated early or to a control group of continued intubation. Randomisation assignments were generated and maintained separately in a sealed, opaque, sequentially numbered envelope [12].

**Figure 1:**
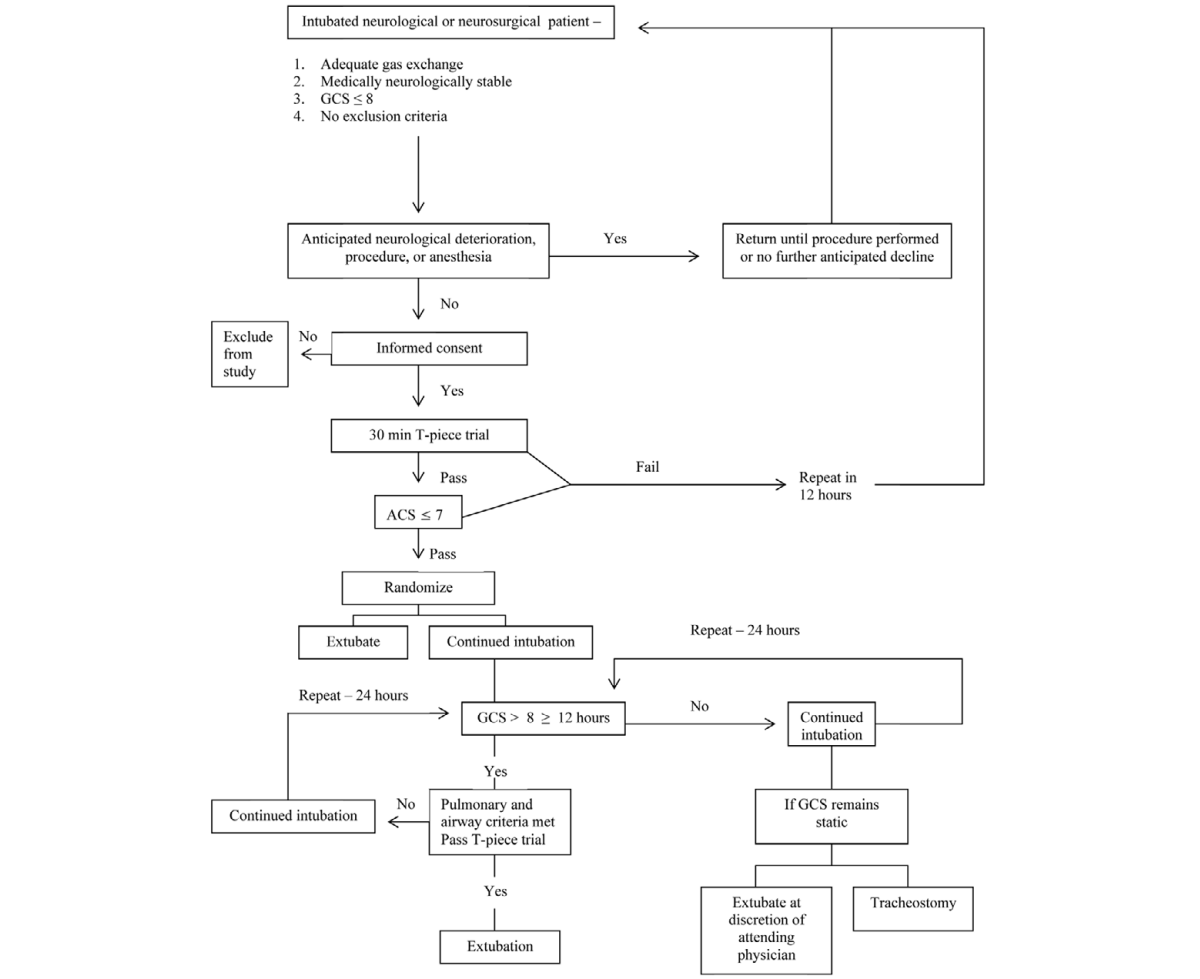
**Algorithm for enrollment and randomisation**. Endotracheal intubated neurological or neurosurgical patients were routinely assessed during morning and evening rounds for eligibility criteria. Consent was obtained from patients' surrogates in medically and neurologically stable patients without anticipated neurological deterioration. Consented patients subsequently underwent a 30 minute T-piece trial and Airway Care Score (ACS) assessment. If the patient failed either assessment, they were re-evaluated in 12 hours. Patients that passed both tests were randomised to early or delayed extubation. Patients randomised to delayed extubation had their Glasgow Coma Score (GCS) reassessed at least every 12 hours. If the GCS improved to more than 8 and they passed the above T-piece and airway reassessments, they were immediately extubated. If the patients neurological status did not improve after several assessments a trial of extubation could still be considered at the discretion of the attending physician to avoid the necessity of placing a tracheostomy.

The control group was reevaluated for possible extubation about every 12 hours during morning and evening rounds using the above protocol. Patients were routinely extubated if the above airway and pulmonary criteria were met and the GCS improved to more than 8 for at least 12 hours. If the patient's neurological examination did not improve with time, a trial of extubation was performed at the discretion of the attending physician to avoid the mandate of tracheostomy placement. Extubation was considered successful if there was no re-intubation within 48 hours. The algorithm for extubation is outlined in Figure 1.

Demographic variables collected at the time of enrollment included age and sex of patients, GCS [13] and the primary cause of neurological deterioration.

GCSs were performed by the attending neurointensivists (EMM, AAR and EFW) in this study. GCSs were obtained before and immediately after extubation of all patients. Patients were given at least a GCS verbal score of one while intubated. Patients that were able to follow midline and appendicular commands but were not oriented to verbal questioning received a verbal score of 3. Patients who were able to follow commands to questions of orientation were given a verbal score of 5. The primary cause of neurological deterioration was categorised into patients with intracerebral haemorrhage, subarachnoid haemorrhage, ischaemic stroke, head trauma and/or brain tumours. The number of patients screened was recorded daily and checked against respiratory therapy records.

Patients were re-intubated if they showed signs of respiratory distress due to an inability to maintain airway patency or respiratory muscle fatigue including: sustained respiratory rate of more than 40 breaths per minute accompanied by accessory muscle use and paradoxical breathing patterns; oxygen saturation of less than 90% for five minutes or partial pressure of oxygen (pO_2_) of less than 60 mmHg on an arterial blood gas; partial pressure of carbon dioxide (pCO_2_) of more than 60 mmHg or a pH of less than 7.3 on arterial blood gas (ABG); loss of pharyngeal of laryngeal tone as noted by gagging or marked sturdor or stridor.

A neurological ICU nurse assessed all patients after extubation for signs of respiratory distress every hour for six hours. A routine ABG was obtained 30 minutes after extubation. The decision to initiate chest physical therapy before and/or after extubation was performed at the discretion of the primary team. The time and reason for any re-intubation was recorded. Any patient participating in the study who was re-intubated was followed but became ineligible for re-enrollment.

Nosocomial pneumonia was defined by traditional criteria as a new or progressive pulmonary infiltrate detected on routine chest radiographs or computed tomography with a temperature higher than 38.5°C, blood leucocyte count of more than 12 × 10^9^/L, and when obtainable tracheal secretions, bronchial washings or blood cultures were consistent with a likely pathogen [14,15].

Routine clinical practice included a chest X-ray and ABG for any signs of respiratory distress or blood cultures for an unexplained fever. Fever was defined as an oral temperature higher than 38.5°C. Follow-up laboratory testing was performed at the discretion of the attending physician. To assess for selection bias the total number of chest images and sputum samples were recorded from enrollment in both the early and delayed extubation groups. Similarly, the total number of days on mechanical ventilation and the number of days after enrollment into the study was recorded for both early and delayed extubation groups.

The primary outcome measure was the functional status of the patient at hospital discharge. The functional status and activity limitations were measured in a blinded fashion by the attending acute rehabilitation service at hospital discharge using the Modified Rankin Scale (MRS) [16] and the Functional Independence Measure (FIM) [17]. Clinicians determining FIM scores were certified in this procedure [17]. Discharge placement categorised as home, rehabilitation or skilled nursing facility was also recorded. Secondary measured parameters included the number of nosocomial pneumonias, re-intubations, and the length of stay in the ICU and hospital.

Morbidity was assessed at hospital discharge by a blinded physician from the department of rehabilitation. Patients were discharged from the ICU at the discretion of the attending physician after a standard prescribed set of discharge criteria was met. The length of stay in the ICU included both time spent in the ICU and the intermediate care area.

A physician not directly involved in the care of the patients provided an analysis for the patients involved in this study after every four patients enrolled using the block randomisation protocol. Enrollment was discontinued if more than three patients needed to be re-intubated or developed nosocomial pneumonias in either the treatment or control group. After analysis of our first four patients, it was discovered that one family requested their family member to not be re-intubated in the event of respiratory or neurological deterioration after randomisation. The medical monitor subsequently required an additional revision that all enrolled patients be eligible for re-intubation. The Mayo Institutional Review board approved the above protocol and the subsequent revision. The above protocol was also reviewed and approved by the Mayo Clinic Intensive Care Unit Committee.

Results are reported as means, standard deviations (SD) and ranges for continuous and ordinal measurements and test for differences in treatment means using two-sided, two-sample student's t-tests assuming unequal variances. We used student's t-tests for these numeric measures because the distributions were not highly skewed, the nonparametric alternatives can suffer from a loss of power at small sample sizes, and we believe the mean is an informative measure of central tendency and an average value for these measures. In sensitivity analyses, inferences were not found to be dependent on the choice of test.

We report the number and percentage of categorical measurements. In analysing treatment differences in a categorical outcome such as re-intubation, we use the chi-squared test without continuity correction when expected cell counts were greater than one and Fisher's exact test in other case [18]. All analyses were performed using R version 2.5.1 statistical software (R Development Core Team. R: A language and environment for statistical computing. R Foundation for statistical computing. Vienna, Austria: 2007 [19]).

## Results

Sixteen patients were randomised between August 2004 and May 2006. Over this time period, 493 patients were screened. Twenty-nine patients met eligibility criteria (5.8% of the screened population). Four families refused randomisation. Nine other patients met initial criteria for enrollment, but in the time it took to reach the families to obtain consent (two to four days), several patients had improved and were extubated, or had worsened from a pulmonary stand point and were no longer eligible. Seven patients were placed on low-dose propofol (Diprivan Astra Zeneca, Pharmaceuticals Wilmington Delaware, USA) for 24 to 48 hours for sedation. All patients had propofol discontinued for at least six hours before enrollment and randomisation. Two delayed extubation patients had propofol reinitiated for less than 24 hours. Nine patients did not receive sedation during their hospitalisation.

Individual patient data is presented in Table 2. Five women and eleven men with an age range from 30 to 93 years were enrolled. Neurological diseases included six head traumas, three tumours, two intracerebral haemorrhages, two subarachnoid haemorrhages and three ischaemic strokes. The GCS at the time of enrollment for all patients ranged between 5 and 8. ACS ranged between 2 and 6. Kappa scores for ACS assessment were good (74) between physician and nurse and excellent (86) between physician and respiratory therapist.

**Table 2:** Individual patient data.

Name	Age/Sex	History	Extubation delay	Total MV days	Initial GCS	Initial ACS	Re-intubate	Nosocomial pneumonia	Days in ICU	Days in hospital	Discharge FIM	Discharge MRS	One-year discharge MRS	Discharge location	One-year discharge location
1	30 F	Tumour	Delayed	9	7	3	No	No	16	40	7	5	3	SNF	Home

2	92 M	Stroke	Early	1	6	5	No	No	2	7	7	5	6	SNF	Dead

3	60 M	Tumour	Delayed	7	7	5	No	Yes	8	20	7	5	6	SNF	Dead

4	86 M	Trauma	Early	3	6	6	No	No	7	26	21	4	2	SNF	Home

5	44 F	ICH	Early	4	7	4	No	No	7	13	26	4	3	Rehab	Home

6	53 F	ICH	Delayed	5	7	6	No	No	8	12	70	4	1	Rehab	Home

7	56 M	Trauma	Delayed	4	7	6	No	No	9	18	23	4	2	Rehab	Home

8	68 M	Trauma	Early	4	7	5	Yes	No	21	21	7	5	6	Withdrawal of care	Dead

9	80 M	Trauma	Early	3	7	5	No	No	5	21	18	5	4	Home	Home

10	51 M	Tumour	Delayed	11	6	3	No	Yes	12	30	18	5	6	Withdrawal of care	Dead

11	36 M	Trauma	Early	6	6	6	No	No	12	25	18	5	3	Rehab	SNF

12	61 F	SAH	Delayed	4	5	5	No	No	4	12	18	5	6	Withdrawal of care	Dead

13	33 F	Stroke	Delayed	17	7	6	No	No	29	44	18	4	2	Rehab	Home

14	64 M	Stroke	Early	5	8	6	No	No	9	14	24	5	6	SNF	Dead

15	93 M	Trauma	Delayed	2	7	5	No	Yes	16	26	23	4	6	SNF	Dead

16	44 F	SAH	Early	4	6	6	No	No	12	47	18	5	3	SNF	SNF

ACS = Airway Care Score; F = female; FIM = Functional Independence Measure; GCS = Glasgow Coma Score; ICH = intracerebral haemorrhage; ICU = intensive care unit; M = male; MRS = Modified Rankin Score; MV = mechanical ventilation; Rehab = rehabilitation facility; SAH = subarachnoid haemorrhage; SNF = skilled nursing facility

There were two possible protocol violations. One patient was enrolled despite a persistent temperature of 38°C orally. At the time of enrollment, this patient had a non-cyclical temperature curve, a negative infectious work up and a hypothalamic tumour. It was agreed by the consultants caring for this patient and the medical monitor that the temperature in this patient was of central origin and did not represent an infectious source. Another patient randomised to early extubation had extubation delayed for four hours to obtain and review repeat head imaging at the request of the primary service.

Patient characteristics for the two groups are presented in Table 3. There were no significant differences between the demographic variables of the two groups; however, the average age of the early extubation group was 10 years older than the delayed extubation group.

**Table 3:** Patient characteristics at enrollment

**Characteristic**	**Early Extubation**	**Delayed extubation**
Number of patients	8	8
Number of women (%)	2 (25.0)	3 (37.5)
Age, years		
Mean (SD)	64.2 (21.1)	54.6 (19.4)
Range	36 to 92	30 to 93
Aetiology		
Tumour	0	3
Stroke	2	1
Trauma	4	2
ICH	1	1
SAH	1	1
Glasgow coma score		
Mean (SD)	6.6 (0.7)	6.6 (0.7)
Range	6 to 8	5 to 7
Airway care score		
Mean (SD)	5.4 (0.7)	4.8 (1.4)
Range	4 to 6	3 to 6

ICH = intracerebral haemorrhage; SAH = subarachnoid haemorrhage; SD = standard deviation.

The total number of mechanical ventilation days was 59 for the delayed extubation group and 30 days for the early extubation group. The average number of days of mechanical ventilation was 7.4 (range = 2 to 17) for the delayed extubation group and 3.8 (range = one to six) for the early extubation group. The average delay in extubation for the delayed extubation group was 3.6 days (range = one to eight). No patient required a tracheostomy. There were 76 chest images obtained in the delayed extubation group and 64 in the early extubation group. Eleven sputum samples were obtained from the delayed extubation group and 10 from the early extubation group.

Patient outcome is presented in Table 4. One patient from the early extubation group was re-intubated and three nosocomial pneumonias were detected in the delayed extubation group. There was one death in the early extubation group and two deaths in the delayed extubation group. There were no unexpected deaths. All deaths occurred after the families or surrogates withdrew medical care due to a poor neurological prognosis. There were no significant differences between treatment groups in the other measured parameters.

**Table 4:** Patient outcomes

**Measurement**	**Early Extubation**	**Delayed extubation**	**P-value**
Glasgow Coma Score at extubation			

Mean (SD)	7.2 (1.0)	8.8 (2.5)	0.16

Range	6 to 9	6 to 13	

Airway Care Score at extubation			

Mean (SD)	5.4 (0.7)	5.2 (1.2)	0.62

Range	4 to 6	3 to 6	

Re-intubation rate			

Number (%)	1 (12.5)	0 (0.0)	1.00

95% confidence interval	1% to 47%	0% to 32%	

Nosocomial pneumonia			

Number (%)	0 (0.0)	3 (37.5)	0.06

95% confidence interval	0% to 32%	14% to 69%	

Stay in ICU, days			0.34

Mean (SD)	9.4 (5.8)	12.8 (7.8)	

Range	2 to 21	4 to 29	

Stay in hospital, days			0.57

Mean (SD)	21.8 (12.1)	25.2 (12.1)	

Range	7 to 47	12 to 44	

Number (%) with a good outcome (Modified Rankin Score less than 4)	0 (0.0)	0 (0.0)	1.00

Modified Rankin Score			

Mean (SD)	4.75 (0.46)	4.50 (0.53)	0.33

Range	4 to 5	4 to 5	

Functional Independence Measure score			0.47

Mean (SD)	17.4 (7.0)	23.0 (20.0)	

Range	7 to 26	7 to 70	

Discharge location, number (%)			1.00

Death, withdrawal of care	1 (12.5)	2 (25.0)	

Skilled nursing facility	4 (50.0)	3 (37.5)	

Rehabilitation facility	2 (25.0)	3 (37.5)	

Home	1 (12.5)	0 (0.0)	

SD = standard deviation.

Although in a larger clinical trial the statistical power would depend on the final study design and analyses specified in the protocol, here we provide sample size estimates to detect differences as larger or larger than those we based on using two-sided, two-sample student's t-tests and a type I error rate of 0.05. The SDs observed in the combined patient group are used for power calculations. The difference in mean MRS was small (0.25) with little variability (SD = 0.5). Therefore, to detect a treatment effect of this size with about 80% power would require 64 patients in each treatment arm. For 90% power, 86 patients would be required for each treatment arm.

The difference in means using the FIM as an endpoint was larger (5.62) as was the SD (14.8 among all subjects). To detect a treatment effect with about 80% power would require 110 patients in each treatment arm. For 90% power, 147 patients in each arm would be required.

The mean ICU length of stay was observed to be 3.4 days shorter for the early extubation group, although the overall SD was 6.8 days. To detect a difference of this size with 80% or 90% power would require sample sizes of an estimated 66 or 88 patients per arm, respectively.

The mean hospital length of stay was reduced by 3.5 days for the early extubation group and the overall SD was 11.8. Due to the greater variability, this end point would require sample sizes of 180 or 240 to obtain 80% or 90% power, respectively.

## Discussion

Traditionally, patients with a GCS of 8 or less would have been intubated because of concerns for airway protection. This procedure arises from a retrospective analysis of the national traumatic coma data bank suggesting that comatose patients not endotracheal intubated had a higher rate of aspiration and worse clinical outcomes [20]. More recent data have similarly supported early intubation in severely brain injured patients [7,21].

The need for initial intubation, however, has been extrapolated to argue that continued intubation is needed in the comatose patient despite a stable neurological condition. In a prospective randomised study, Namen and colleagues reported an incremental increase in successful extubations in neurosurgical patients with an increasing GCS. They found a 61% extubation failure rate for patients with a GCS of 8 or less [22]. However, in a large prospective observational analysis, Coplin and colleagues reported an increase in nosocomial pneumonias, increased length of stay and worse outcomes in patients who had extubation delayed over concerns of compromised consciousness [11]. Multiple calls for randomisation have been challenged because of a concern that randomisation may not be feasible secondary to ingrained suppositions as to who can be safely extubated. (W. Coplin, personal communication). The results of this trial argue strongly that randomisation of severely brain injured patients to early and delayed extubation is both technically feasible and safe to perform.

Patients in the neurological ICU may remain intubated for treatment of their primary neurological illness including sedation for control of intracranial hypertension and optimisation of cerebral blood flow in the treatment of cerebral vasospasm and ischaemic stroke. We were careful to delineate a population of patients that were beyond the acute phase of brain injury and were believed unlikely to deteriorate from secondary neurological causes. Patients that were intubated for sedation, therapeutic hyperventilation or were deemed to be at risk for the development of cerebral vasospasm that would require the acute management of cerebral perfusion pressure were excluded until these risks were considered to no longer be present. This was based on the clinical judgement of the authors, but will need to be more objectively defined in a larger study. This may include documentation of adequate cerebral perfusion without vasopressor support, lack of a need for osmotic treatment of intracranial hypertension and decreasing transcranial Doppler ultrasound flow velocities in patients with subarachnoid haemorrhage.

The requirement of a low modified ACS ensured that all enrolled patients had minimal airway secretions. The presence of a quantifiable spontaneous strong cough and minimal respiratory secretions has been shown to have a strong correlation with extubation success [23-26]. By requiring good control of airway secretions for enrollment, we were able to isolate a population of patients that remained intubated solely because of their level of consciousness. We were thus able to address a single question of whether a GCS of 8 or less should preclude extubation.

We chose to use a modified ACS using cough to suctioning rates as opposed to a quantifiable measure of cough flow rates. We believe that this method was simple to use and reproducible across ICU personnel. The good to excellent correlations between users verified its utility but may require training and more standardisation for a larger study.

The timing and placement of tracheostomies in this population is controversial. Some authors have advocated early placement of tracheostomies in patients with a decreased level of consciousness [27-29]. Our methodology allowed us the option to consider a trial of extubation in the delayed extubation group before requiring placement of a tracheostomy. This option to some degree reflects an institutional bias against unnecessary tracheostomy placement and was required by our ICU committee.

The average delay in extubation was 3.6 days in the delayed extubation group. The ICU length of stay and hospital length of stay was increased by 3.4 days in the delayed extubation group suggesting that extubation delay was the primary source of increased length of stay. Although a wide range of variations in ICU length of stay, hospital length of stay and extubation delay existed, review of our respiratory data did not reveal an increase in suctioning frequency or respiratory care for patients that were extubated early.

Similarly, we do not think that selection bias played a significant role in the detection of nosocomial pneumonia given that a similar number of sputum samples were evaluated and there was only a slight increase in the number of chest images obtained in the delayed extubation group.

One patient in the early extubation group required re-intubation. The re-intubation was likely to be iatrogenic caused by epistaxis after placement of a nasal airway. We therefore believe that our limited results suggest early extubation is most likely to be safe to perform in this population.

The limited number of patients in this study precluded statistical analysis with adequate power to make any definitive statements but did allow for power estimates for a larger study. We decided to base these estimates on two functional measures of neurological outcome. The MRS is the most reliable and commonly used functional measure for long-term neurological outcome. A relatively small sample size was required for a larger study using the MRS due to the noted low variability in this sample size. This may reflect the relative insensitivity of this measure. The FIM is the most sensitive evaluation to detect a small difference in outcome. A larger sample size was required using the FIM; however, the differences noted remained small and may not be of functional significance.

We chose to follow patient outcome as the primary outcome for this study. Although secondary outcomes could be used as the primary outcome, with presumably smaller numbers needed to test superiority, this would still leave the question of whether the intervention affected the outcome. We therefore believe that there is an advantage to designing a non-inferiority trial, which would assume non-inferiority for neurological outcome but test superiority for secondary measures. For example, a trial with less than 100 subjects per arm would have high power to establish that early extubation did not negatively impact MRS (assuming the largest acceptable difference in MRS was 0.75 points and early extubation did not increase the mean MRS by more than 0.50 points) and shortened ICU length of stay. Larger numbers, however, would be required if overall hospital length of stay was used as the secondary endpoint for an equivalence trial. This trial would have obvious economic implications.

The low percentage of screened patients who were eligible for enrollment reflects our strict inclusion criteria, and the demographics of our unit with a high number of postoperative patients and relatively few severe head traumas. We did, however, include a broad spectrum of neurological illnesses. A larger study will require multiple sites with variable patient populations.

## Conclusion

In conclusion, randomisation of severely brain injured patients to early or delayed extubation did not identify any safety concerns and is feasible. The results of a larger multicentre trial will have significant implications for the ICU care of brain injured patients.

## Key messages

• Randomisation of brain injured patients to early extubation appears to be safe and feasible.

• Extubation decisions can potentially be made based on randomised trials and not on observational studies alone.

• Larger randomisation studies are needed to further delineated extubation criteria in this population.

## Abbreviations

ABG: arterial blood gas; ACS: Airway Care Score; FiO_2_: fraction of inspired oxygen; FIM: Functional Independence Measure; GCS: Glasgow Coma Score; ICU: intensive care unit; MRS: Modified Rankin Score; PaO_2_: partial pressure of arterial oxygen; PCO_2_: partial pressure of carbon dioxide; PO_2_: partial pressure of oxygen; SD: standard deviation.

## Competing interests

This research was supported by the Mayo Clinic Department of Neurology discretionary funds. The authors declare they have no competing interests.

## Authors' contributions

EMM conceived of the study, participated in its design and coordination, enrolled patients, and drafted and rewrote the manuscript. AAR participated in the design and coordination of the study, enrolled patients and aided in the drafting of the manuscript. EFMW participated in the design and coordination of this study, enrolled patients and aided in the drafting of the manuscript. AWB performed the FIM, MRS and aided in the drafting of the manuscript. WDF enrolled patients and aided in the drafting of the manuscript. VHL enrolled patients and aided in the drafting of the manuscript. SAW developed and performed the statistical analysis for the study, and aided in the drafting and revision of the manuscript. MTK participated in the coordination and data acquisition of patients and aided in the drafting of the manuscript. DRB participated in the coordination and data acquisition of patients and aided in the drafting of the manuscript. FXW participated in the coordination and data acquisition of patients and aided in the drafting of the manuscript. TKR participated in the coordination and data acquisition of patients and also aided in the drafting of the manuscript. RDH participated in the design and coordination of the study.
